# Liposomal hydrogel-based oral vaccine delivery for targeted induction of intestinal mucosal immunity

**DOI:** 10.1016/j.mtbio.2026.102975

**Published:** 2026-02-28

**Authors:** Zhiwei Li, Baochao Fan, Chengcheng Ouyang, Mi Hu, Xu Song, Guoguang Chen, Yiwen Lou, Huajun Yang, Dongmei Sun, Bin Li, Lili Ren

**Affiliations:** a School of Pharmacy, Nanjing Tech University, Nanjing, 211816, China; b Institute of Veterinary Medicine, Jiangsu Academy of Agricultural Sciences, Key Laboratory of Veterinary Biological Engineering and Technology Ministry of Agriculture; Jiangsu Key Laboratory for Food Quality and Safety-State Key Laboratory Cultivation Base of Ministry of Science and Technology, Nanjing, 210014, China; c Jiangsu Co-Innovation Center for the Prevention and Control of Important Animal Infectious Disease and Zoonose, Yangzhou University, Yangzhou, 225009, China

**Keywords:** Sodium alginate, Mannose-modified liposome, Oral vaccine, PEDV, Mucosal immunity, Systemic immunity

## Abstract

Oral vaccines have attracted considerable attention due to their advantages of convenient administration and ability to induce mucosal immunity. However, unfavorable conditions such as the gastrointestinal barrier and acidic environment constrain their immunogenic efficacy. To address these issues, a novel oral vaccine delivery platform has been developed, in which mannose-decorated liposomes are complexed with antigen and retinoic acid, then enveloped by a thiolated alginate gel microsphere (MLip@Gel). Mannose-modified liposomes adsorb porcine epidemic diarrhea virus (PEDV) through electrostatic interactions, targeting intestinal macrophages and enhancing uptake. Thiol-modified sodium alginate is used as a gel shell, preventing PEDV from being destroyed in the stomach and promoting retention in the intestines. Retinoic acid (RA) facilitates the differentiation of cells that promote secretory IgA production. The delivery system maintain stability under acidic conditions, while decomposing at pH ≥ 6.8 to release the antigen. Experiments on mice demonstrate that PR-MLip@Gel can induce a high level of α4β7^+^CCR9^+^ cell activation and IgA level, compared to PEDV(IM) group. Systemic responses, such as IgG and CD4^+^/CD8^+^ T, B cells, are also significantly increased. The challenge experiments demonstrate that piglets immunized with PR-MLip@Gel exhibit better protection against PEDV infection, as PR-MLip@Gel can simultaneously induce robust mucosal immunity and humoral immunity. These findings suggest that the oral vaccine effectively induces strong mucosal and systemic immune responses, offering a promising strategy for developing oral vaccines against intestinal infectious diseases.

## Highlights

• This study developed an oral intestinal mucosal vaccine delivery system (PR-MLip@Gel).
• It resists degradation by gastric acid and enzymes, enabling sustained antigen release in the intestine.
• It effectively stimulates mucosal immunity and enhances systemic immunity.
• The prepared oral vaccine demonstrates good biocompatibility and safety.

## Graphical abstract

**Figure:**
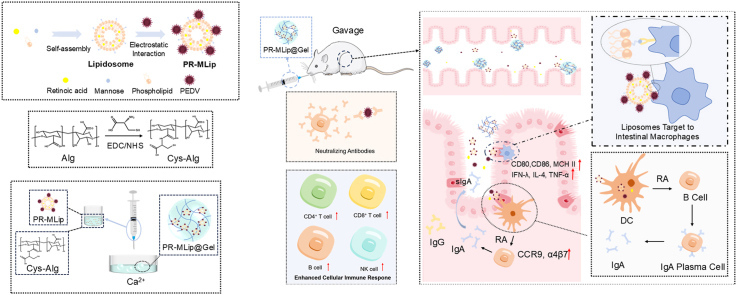

## 1 Introduction

Viral infections pose a major threat to global health, and vaccination remains the most effective prevention strategy [1,2]. While vaccines typically induce serum IgG responses, the immune activation, particularly at mucosal surfaces, can be limited [3,4]. Mucosal immunity, characterized by secretory IgA (sIgA), plays a critical role in neutralizing pathogens at entry points [5,6].

Oral vaccines can induce mucosal immunity and are easy to administer, but face challenges such as gastric acidity and enzymatic degradation [[7], [8], [9]]. Effective oral delivery systems must protect antigens from the stomach, penetrate intestinal mucus, facilitate epithelial absorption, and activate antigen-presenting cells (APCs).

To address these barriers, strategies like using protective coatings (e.g., HPMCP or sodium alginate) have been employed, though with risks of premature antigen release [10]. Particle shape and size influences uptake; rod-shaped carriers have shown superior intestinal absorption over spherical ones [11]. Additionally, ligands like β-glucan promote APC activation via receptors such as dectin-1 on M cells [12,13]. However, many studies focus on optimizing single aspects, lacking integrated solutions.

Liposomes are promising oral vaccine carriers due to their customizable surfaces and mucosal permeability [[14], [15], [16]]. Their membranes-fusing capablities facilitate cellular uptake and APC [17]. Thiolated sodium alginate, as a protective gel shell, protects antigen from acid and improves intestinal adhesion, prolonging retention and interaction time [[18], [19], [20]]. To enhance immunogenicity, mucosal adjuvants like retinoic acid (RA), a vitamin A metabolite, can promote IgA-secreting cell differentiation and regulate intestinal immunity [[21], [22], [23], [24], [25]].

Based on these findings, this study used porcine epidemic diarrhea virus (PEDV) as the antigen and designed an adjuvant system based on cationic liposomes. PEDV is an enteric coronavirus that causes highly contagious acute intestinal disease with a high mortality rate [26]. Cationic liposomes were modified with mannose (MLip) for targeting intestinal macrophages, and co-loaded with RA and attenuated PEDV (PR-MLip). To enhance protection, PR-MLip was encapsulated with thiolated sodium alginate, forming stable liposomal gel microspheres (PR-MLip@Gel) for gastric resistance and prolonged intestinal retention. This system aims to synergistically enhance mucosal and systemic immune responses and offers a promising strategy for next-generation oral vaccines.

### 1.1 Materials

Sodium alginate was purchased from Aladdin, Co., Ltd. (Shanghai, China). L-cysteine hydrochloride anhydrous, sodium hydroxide (NaOH), 1-(3-dimethy laminopropyl)-3-ethylcarbodiimide hydrochloride (EDC), N-hydroxysuccinimide (NHS), etc. were purchased from Macklin Biochemical Co., Ltd. (Shanghai, China). Penicillin/streptomycin solution, fetal bovine serum (FBS), DMEM medium, RPMI 1640 medium, and phosphate buffered saline (PBS) were purchased from Nanjing Senberga Biotechnology Co., LTD. (Nanjing, China). IFN-γ, TNF-α, and IL-4 ELISA kits were purchased from Nanjing Liangwei Biotechnology Co., LTD. (Nanjing, China). Unless otherwise stated, all reagents were of analytical grade and were purchased from the supplier.

### 1.2 Animals

Female BALB/c mice were originally acquired from Beijing Vital River Laboratory Animal Technology Co., Ltd. (Beijing, China). The animal experiments were complied with the ARRIVE guidelines and carried out in accordance with EU Directive 2010/63 for the protection of animals used for scientific purposes, approved by the Animal Ethical and Welfare Committee of Nanjing Tech University (approval ID: lACUC-20240527-02).

### 1.3 Preparation and characterization of PR-MLip@Gel

#### 1.3.1 Preparation and characterization of cysteine modified sodium alginate (Cys-Alg)

Alginate solution (2%, 20 mL) was activated with EDC (177.39 mg) and NHS (106.50 mg) at room temperature for 45 min. L-cysteine (145.86 mg) was added, pH was adjusted to 5-6 using 0.1 M NaOH, and reacted for 12 h. Dialysis was performed sequentially with HCl (5 mM then 1 mM; 8000 - 14,000 kDa). After pH adjustment to 4, samples were lyophilized and stored at 4 °C. The reaction was processed in the dark. Samples were analyzed using Fourier Transform Infrared Spectrometer (FTIR) and ^1^H NMR spectroscopy (Bruker, Germany); the thiol content was determined via DTNB assay.

#### 1.3.2 Preparation and characterization of PR-MLip

Phosphatidylcholine, DC-cholesterol, Man-PEG-DSPE, and RA (1 mg/mL each in ethanol; 15:5:0.3:0.6, w/v) were rotary-evaporated (40 °C) to form films. Films were hydrated in PBS (3 mL, 40 °C, 1 h), sonicated (5 min), and filtered (0.45 μm × 3) to obtain RA-MLip. Blank MLip excluded RA. PR-MLip was prepared by incubating liposomes (15 mg) in PBS (1 mL) with PEDV (250 μL, 10^5^ TCID_50_/mL) overnight. The size, zeta potential and morphology were analyzed by a Malvern Zetasizer Nano ZSE and emission scanning electron microscope (SEM, Quanta FEG 250, FEI, USA).

#### 1.3.3 Preparation and characterization of PR-MLip@Gel

PR-MLip (37.50 mg) was mixed with Cys-Alg (5 mL, 1.5%, w/v) and injected (5 mL/min) into CaCl_2_ (3%, 50 mL). After crosslinking for 20 min, gels were lyophilized and cryo-fractured for SEM analysis. FITC-OVA-labeled gels were imaged by fluorescence microscopy.

### 1.4 *In vitro* degradation and release assays

MLip@Gel degradation was assessed in SGF (pH 1.2) and SIF (pH 6.8, enzyme-free). Morphological changes (0–6 h) and swelling properties were recorded. Release profiles of PR-MLip and PR-MLip@Gel were determined in SIF (24 h) followed by SGF (2 h). RA and OVA release were quantified via HPLC and BCA, respectively.

### 1.5 *In vitro* intestinal adhesion assay

MLip, MLip@Gel (Alg), and MLip@Gel (Cys-Alg) were applied to porcine intestinal segments (5 cm), rinsed with PBS for 3 times, and adherent microspheres counted.

### 1.6 *In vivo* biodistribution assays

FITC, FITC-MLip, and FITC-MLip@Gel were administered by gavage to mice at a dose of FITC (80 mg/kg). Small intestines were excised (0–24 h) and imaged (VISQUE InVivo Smart-LF). Intestinal loops (porcine) injected with fluorescent samples were incubated in Krebs-Ringer buffer (37 °C). FITC permeation was measured fluorometrically. RAW264.7 cells and dendritic cells were incubated (4 h) with FITC-OVA formulations (50 μg/mL), DAPI-stained, and imaged by confocal microscopy (LSM 880, Zeiss, Germany). Mannose competition (Man + FITC-MLip) was tested in RAW264.7 only.

### 1.7 Animal immune experiments

Female BALB/c mice (6–8 weeks, 18–22 g) were randomised into six groups (*n* = 6). Five groups were orally immunized with PBS (200 μL), PEDV (TCID_50_ 10^5^/200 μL), RA, MLip@Gel (200 μL), or PR-MLip@Gel (200 μL). The sixth group was injected intramuscularly with PEDV (2 × 10^5^ TCID_50_) mixed 1:1.5 (v/v) with aluminium hydroxide adjuvant and brought to 200 μL. Doses were given on days 0, 14 and 28. Feces were collected pre-study and 48 h after each dose. Mice were euthanized on day 42 and organs harvested for H&E histology. All procedures were approved by the Nanjing Tech University Animal Ethics Committee (IACUC-20240527-02) and complied with relevant guidelines and regulations. Mice were sacrificed 96 h post-prime to harvest bone marrow cells [27]. The cells were then stained with PE-anti-CD80, FITC-anti-CD86, and APC-anti-MHC II for 30 min at 4 °C in the dark and analyzed by flow cytometry. PEDV-specific IgG and IgA in the serum, feces and intestinal lavage fluid of the mice were measured by ELISA, using the PEDV spike protein as the antigen, and neutralizing titres were determined on Caco-2 cells using the Karber method. Splenocytes were stimulated with concanavalin A (10 μg/mL, 72 h); The levels of cytokines (TNF-α, IFN-γ, and IL-4) were quantified using ELISA kits following the manufacturer's instructions. For phenotype analysis, isolated splenic lymphocytes were divided into three aliquots and stained with specific antibody cocktails for 30 min at 4 °C in the dark: (i) FITC-anti-CD3e, PE-anti-CD4, and APC-anti-CD8a for T cell subsets; (ii) FITC-anti-CD19 and PerCP-Cy5.5-*anti*-B220 for B cells; and (iii) FITC-anti-CD3e and APC-anti-CD49b for NK cells. Intestinal lymphocytes were stained with APC-anti-α4β7 and PE-anti-CCR9. All cells were washed twice with PBS before flow cytometric analysis.

### 1.8 Protective-efficacy challenge in piglets

In the immunization and challenge experiments, 12 7-day-old, PEDV negative piglets were randomly assigned to four groups (Control, PBS + PEDV, PEDV(IM) + PEDV and PR-MLip@Gel + PEDV) and housed in different rooms. Animals in the PR-MLip@Gel + PEDV and PEDV(IM) + PEDV groups were orally gavaged and intramuscular injected with 2 mL of the corresponding formulation on Days 0 and 14, respectively. And the PBS + PEDV group received the same volume of sterile PBS. Serum samples were collected from each piglet before at 28 day.

The viral challenge experiments were conducted at 28 day. The piglets were challenged with the AH2012/12 strain of PEDV (GenBank accession: KU646831) via the oral route of inoculation (2.0 × 10^7.0^ TCID_50_ per piglet) [28]. Body weights and faecal swabs were recorded daily. Intestinal tissue and intestinal contents samples were collected for pathological evaluation and the quantification of the PEDV RNA load. The intestinal tissue and intestinal contents samples collected were also used to detect the specific antibodies. All the piglet experiments were performed according to the protocol approved by the Experimental Animal Ethics Committee (NKYVET 2015–0127) of Jiangsu Academy of Agricultural Sciences, China. All efforts were made to minimize animal suffering and to reduce the number of animals used.

### 1.9 Statistical analysis

Graphics and statistical analysis were performed using Prism GraphPad 8.0 or Origin software. Unless otherwise specified, all experiments were repeated three times (*n* = 3), and the data were expressed as mean ± SD. Statistical differences among groups were determined using one-way ANOVA followed by Tukey's multiple comparisons test. P < 0.05 (∗) was considered significant, P < 0.01 (∗∗) was highly significant, and P < 0.001 (∗∗∗) was extremely significant.

## 2 Results and discussion

### 2.1 Successfully prepared PR-MLip@Gel

**Fig. 1:**
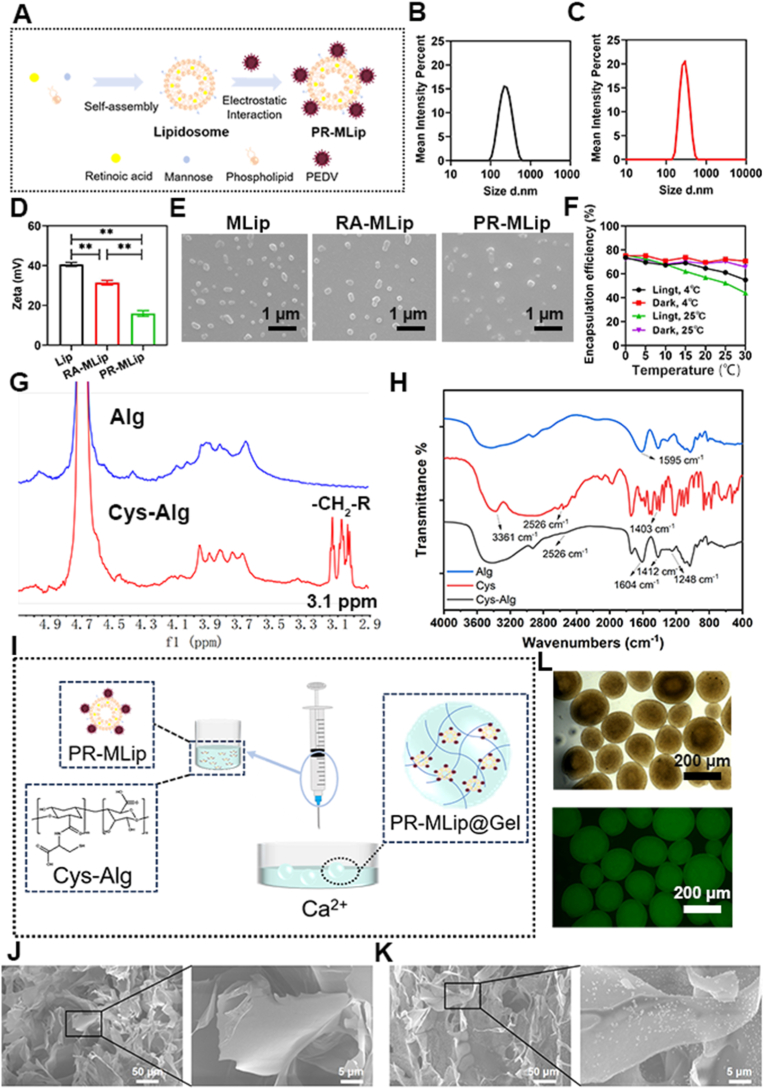
Preparation and characterization of PR-MLip@Gel. (A) Schematic diagram of PR-MLip preparation. (B) Particle size distribution of MLip. (C) Particle size distribution of PR-MLip. (D) Zeta potentials of MLip, RA-MLip, and PR-MLip. (E) SEM analysis (MLip, RA-MLip, and PR-MLip from left to right, scale bar: 1 μm). (F) Stability of PR-MLip under different storage conditions. (G) Nuclear magnetic resonance hydrogen spectroscopy of Alg and Cys-Alg. (H) Infrared spectra of Alg, Cys, and Cys-Alg. (I) Schematic of the preparation of PR-MLip@Gel. (J) SEM analysis of gel microspheres. (K) SEM analysis of MLip@Gel. (L) Inverted fluorescence microscope image of FITC-labeled liposome gel microspheres (images viewed from top to bottom in bright field and dark field, scale bar: 200 μm). Data are expressed as mean ± SD (*n* = 3).

Single liposomes and hydrogels face challenges in oral delivery, including gastric burst release, poor intestinal mucosal retention, and low intestinal absorption [29,30]. We developed cationic mannose-modified liposome gel microspheres for targeted intestinal delivery (Fig. 1A). RA-loaded liposomes (RA-MLip) were prepared via filming-rehydration. Negatively charged attenuated PEDV was adsorbed electrostatically to form PR-MLip.

DLS revealed particle sizes of 215.70 ± 5.86 nm (MLip), 236.43 ± 8.07 nm (RA-MLip), and 318.23 ± 2.61 nm (PR-MLip) (Fig. 1B, C and S1). PDIs were 0.167 ± 0.016 (MLip), 0.170 ± 0.014 (RA-MLip), and 0.269 ± 0.039 (PR-MLip), confirming uniform dispersions. Zeta potential decreased significantly after RA and PEDV loading (Fig. 1D). SEM showed larger sizes than DLS, likely due to flattening during drying (Fig. 1E). RA-MLip exhibited encapsulation efficiency of 78.18 ± 1.52% and drug loading of 2.35 ± 0.05%. Liposomes improved RA's solubility and stability under dark storage at 4 °C, encapsulation efficiency remained 70–75% for 30 days. Light exposure reduced efficiency by 18.67% (4 °C) and 31.40% (25 °C) (Fig. 1F). No significant changes in size, PDI, or zeta potential indicated good stability of RA-MLip. These physicochemical traits are critical for oral delivery: particles between 200 and 350 nm penetrate intestinal mucus efficiently yet remain large enough to avoid paracellular clearance, while a mildly positive surface favours electrostatic adhesion to epithelial and immune cells.

To ensure gastric stability, calcium alginate (Ca-Alg) was selected as the shell material due to its acid stability and moderate adhesiveness. However, its weak intestinal adhesion and short residence time necessitated modification. Cysteine-modified alginate (Cys-Alg) was synthesized via EDC/NHS-mediated amidation to enhance mucoadhesion through sulfhydryl groups, prolonging intestinal residence and improving bioavailability [19]. Successful grafting was confirmed by ^1^H NMR (New shifts at 3.08 and 3.13 ppm; Fig. 1G) and FTIR spectrum (characteristic peaks at 1248, 1412, 1604 cm^−1^ and 2526 cm^−1^; Fig. 1H). DTNB quantification showed 5.77 ± 0.24 mol% sulfhydryl content. PR-MLip was mixed with Cys-Alg and injected into CaCl_2_ to form PR-MLip@Gel microspheres with diameters of 200 - 250 μm (Fig. 1I–S2). SEM revealed porous network structures in both blank and liposome-loaded gels (Fig. 1J), with uniform granular distributions preliminarily identified as MLip(Fig. 1K). Fluorescence microscopy of FITC-labeled microspheres confirmed homogeneous liposome distribution within the gel matrix (Fig. 1L).

### 2.2 PR-MLip@Gel exhibits resistance to gastric degradation and efficient intestinal release

**Fig. 2:**
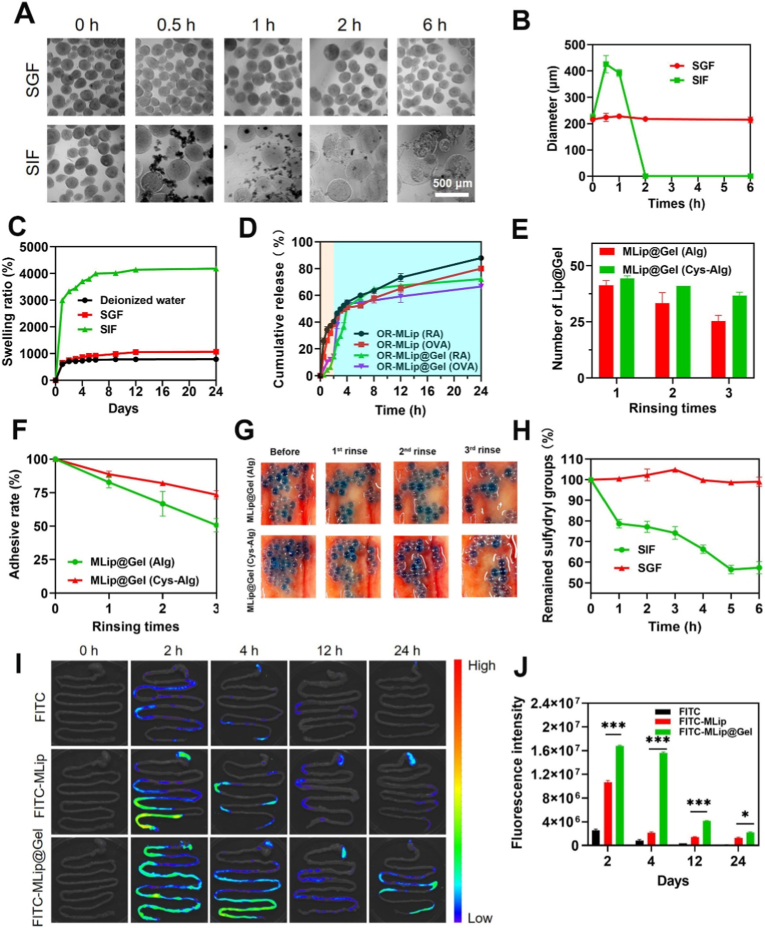
Resistance of MLip@Gel to the gastrointestinal environment and retention in the gut. (A) Representative image of the degradation process of MLip@Gel in SGF and SIF (Scale bar: 500 μm). (B) Size change curve of MLip@Gel in SGF and SIF. (C) The swelling ratio of MLip@Gel in water, SGF, and SIF. (D) Simulated RA and PEDV release curves of PR-MLip and PR-MLip@Gel under gastrointestinal fluid conditions. (E) Statistics of the number of microspheres at MLip@Gel (Alg) and MLip@Gel (Cys-Alg) after washing. (F) MLip@Gel (Alg) and MLip@Gel (Cys-Alg) adhesion comparison. (G) Images of residual MLip@Gel (Alg) and MLip@Gel (Cys-Alg) on the surface of the small intestine before and after three washes. (H) Curves of sulfhydryl content of Cys-Alg in SGF and SIF. (I) *In vitro* imaging of the distribution of FITC, FITC-MLip, and FITC-MLip@Gel in the gastrointestinal tract after oral administration. (J) Quantification of FITC, FITC-MLip, and FITC-MLip@Gel. Data are expressed as mean ± SD (*n* = 3).

Gastrointestinal transit typically requires 1-2 h in stomach and 1-6 h in small intestine [31]. MLip@Gel demonstrated gastric stability in simulated gastric fluid (SGF, pH 1.2), maintaining morphology after 6 h (Fig. 2A). In simulated intestinal fluid (SIF, pH 6.8), structure collapse occurred within 1 h. The diameter of MLip@Gel remained stable in SGF but increased in SIF prior to collapse (Fig. 2B), the diameter of MLip@Gel initially increased significantly before the gel structure collapsed and became non-spherical. Swelling ratios were 785.02 ± 4.77% in water and 1065.85 ± 6.55% in SGF, while SIF induced rapid swelling to 4181.89 ± 95.56%. (Fig. 2C), demonstrating strong tolerance to SGF.

OVA served as a model antigen for PEDV in release studies. As shown in Fig. 2D, OR-MLip@Gel exhibited significantly lower RA and OVA release in SGF than OR-MLip, with cumulative release under 15% at 2 h versus 40.45 ± 0.36% (RA) and 38.56 ± 2.21% (OVA) for OR-MLip, confirming effective gastric retention by the gel shell. Upon SIF transfer, OR-MLip@Gel release accelerated, reaching 51.28 ± 0.59% (RA) and 54.08 ± 2.58% (OVA) within 2 h, comparable to OR-MLip (54.83 ± 1.16% RA, 51.06 ± 1.19% OVA), followed by sustained release. Final cumulative release reached 72.17 ± 0.31% (RA) and 66.61 ± 0.95% (OVA), lower than OR-MLip (87.96 ± 0.44% RA; 80.02 ± 1.73% OVA), attributable to diffusion hindrance by the gel network. This oral delivery system is able to protect the drug in the highly acidic gastric environment, preventing its release in the stomach and ensuring that release occurs only after the formulation reaches the intestine.

PR-MLip@Gel thus demonstrates gastric stability with minimal drug leakage and intestinal-responsive release, confirming its targeted delivery.

### 2.3 Retention of liposome gel microspheres in the intestine is enhanced

Intestinal retention is crucial for sustaining local drug concentrations. To assess the mucoadhesive effect of Cys-Alg, an *in vitro* adhesion assay was performed on fresh porcine small intestinal epithelium [32,33]. MLip, MLip@Gel (Alg), and MLip@Gel (Cys-Alg) were placed on the mucosa of the upright intestinal mucosa. MLip slid off immediately, while both gel-based microspheres adhered for extended periods (Fig. S3). MLip@Gel (Cys-Alg) showed greater microsphere retention and a smaller spread area after 120 min. When dispersed in PBS and applied to the intestinal surface, both gel groups retained some microspheres after PBS rinses. Adhesion rates were 50.67 ± 5.03% for MLip@Gel (Alg) and 73.33 ± 3.05% for MLip@Gel (Cys-Alg), indicating significantly enhanced adhesion due to cysteine modification (Fig. 2E–G). This enhancement is likely driven by disulfide bond formation within the thiolated polymer, which increases viscosity and mucoadhesion [34]. Disulfide formation was studied by measuring the remaining sulfhydryl groups (Fig. 2H), which remained stable in SGF but decreased to 67.9% after 1 h in SIF.

*In vivo* biodistribution was assessed using FITC-labeled OVA as a drug model. Compared to FITC and FITC-MLip, the FITC-MLip@Gel showed stronger and more sustained fluorescence in the gastrointestinal tract (Fig. 2I and J). While fluorescence of the FITC group disappeared by 12 h, FITC-MLip@Gel retained detectable fluorescence up to 24 h. These results indicate that the hydrogel encapsulating the liposomes exhibits strong intestinal mucoadhesive properties, thereby prolonging the residence time of the antigen in the intestine.

### 2.4 PR-MLip@Gel enhances Drug Absorption and Targeted Delivery

**Fig. 3:**
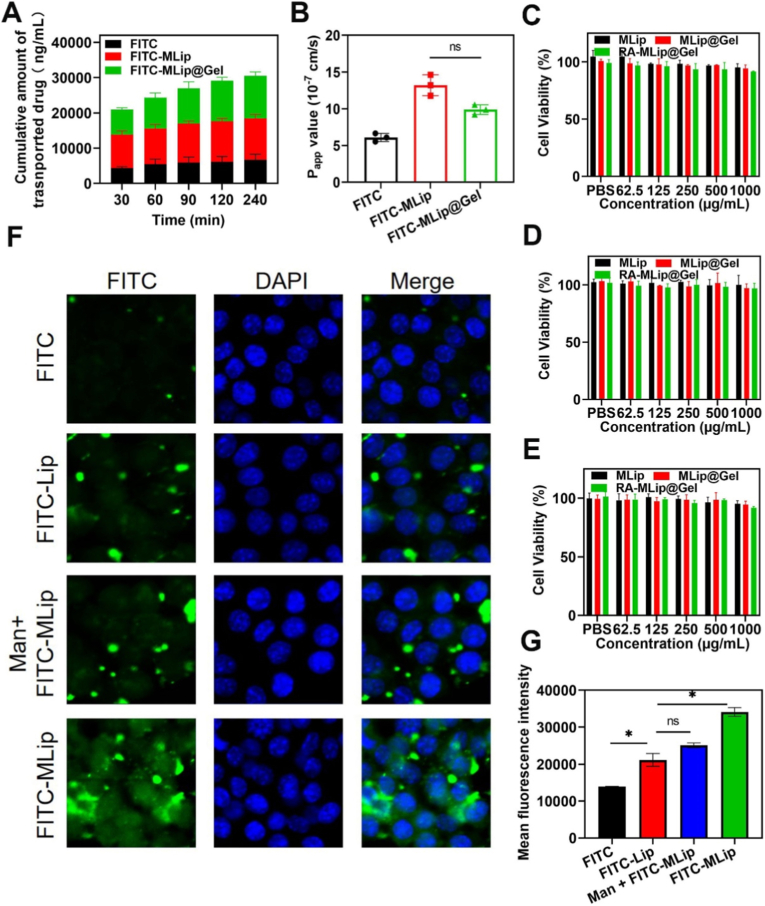
Drug Absorption and Targeted Delivery. (A) Cumulative permeability of FITC, FITC-MLip, and FITC-MLip@Gel across the small intestine. (B) P_app_ values of FITC, FITC-MLip, and FITC-MLip@Gel in the small intestine. Cell Viability of Caco-2 cells (C), RAW264.7 cells (D), and DCs (E) at different MLip, MLip@Gel, and RA-MLip@Gel concentrations treatment for 24 h. (F) Confocal images of RAW264.7 cells uptake (Magnification: 40 × ). (G) Fluorescence quantitative analysis of uptake by RAW264.7 cells. Data are expressed as mean ± SD (*n* = 3).

Improved mucus permeation and intestinal absorption enhance drug action [35]. To evaluate liposome-mediated delivery, FITC-labeled OVA was used as antigen mimic to evaluate *in vitro* intestinal permeation across FITC, FITC-MLip, and FITC-MLip@Gel groups. All showed time-dependent permeation (Fig. 3A and B), with FITC-MLip@Gel showed the highest cumulative permeation (P_app_ = 9.90 × 10^−7^ cm s^−1^), 1.79-fold greater than FITC. Although initial accumulation was lower than FITC-MLip, FITC-MLip@Gel later surpassed it, likely due to swelling of the Cys-Alg hydrogel. The delayed but higher permeation is attributable to gradual swelling of the thiolated gel, which converts the microsphere into a depot continuously feeding liposomes to the epithelium. This suggests that positively charged liposomes facilitate mucus penetration and absorption. Cytotoxicity tests in Caco-2, RAW264.7, and DCs showed >90% viability across all concentrations (0–1 mg/mL) (Fig. 3C–E), indicating low cytotoxicity.

Confocal imaging using FITC-labeled OVA (Fig. 3F and G). showed stronger fluorescence in liposomal groups versus free FITC, indicating enhanced internalization. FITC-MLip uptake surpassed unmodified FITC-Lip, while pre-treatment with mannose (Man + FITC-MLip) reduced uptake, confirming mannose receptor-mediated targeting. Similar patterns were observed in DCs (Figs. S4–S5), likely due to mannose receptor expression. These findings confirm that mannose-modified liposomes enable targeted delivery to intestinal APCs, supporting their potential in mucosal immunotherapy.

### 2.5 PR-MLip@Gel enhances antigen presentation and immune activation

Antigen-specific immunity relies on APC activation and immune cell recruitment. Adjuvants enhance this by promoting DC and macrophage maturation, marked by CD80^+^, CD86^+^, and MHC II + expression [36].

**Fig. 4:**
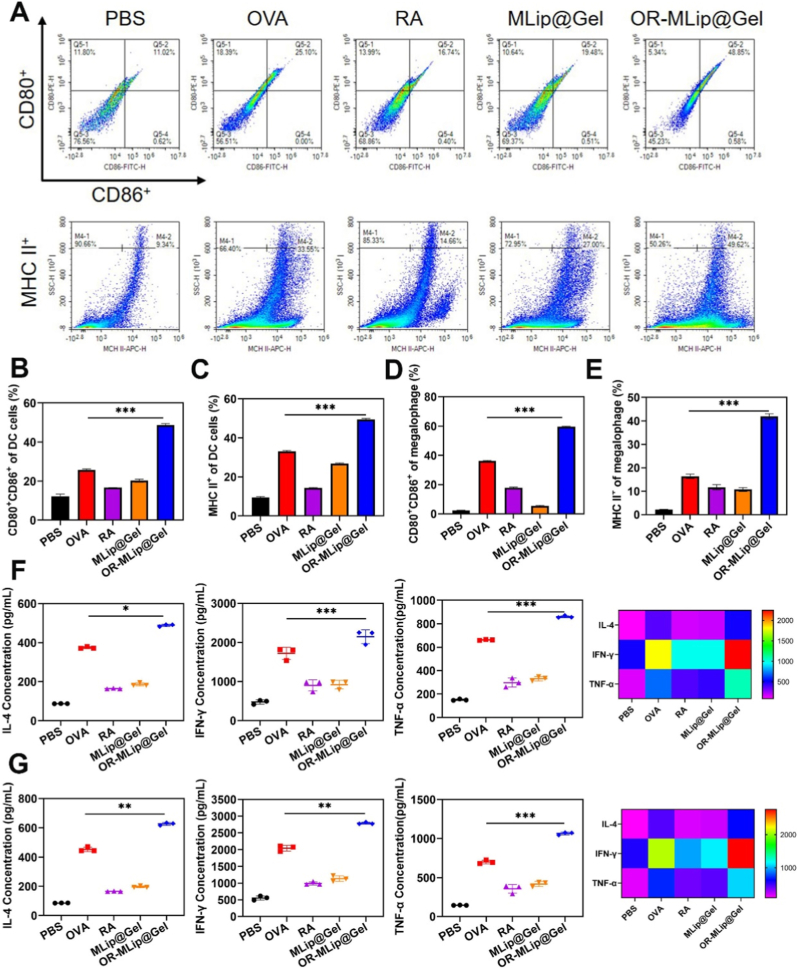
*In vitro* immune activation of the vaccine. (A) Representative flow cytometry plot of CD80^+^CD86^+^ and MHC II^+^ on DCs. Expression of CD80^+^CD86^+^ (B), and MHC II^+^ (C) on DCs. Expression of CD80^+^CD86^+^ (D), and MHC II^+^ (E) on RAW264.7 cells. (F) The expression of IL-4, IFN-γ and TNF-α cytokines in DCs was stimulated by *in vitro* immunization. (G) Cytokine expression of IL-4, IFN-γ and TNF-α in RAW 264.7 cells were stimulated by *in vitro* immunization.

To evaluate the adjuvant potential of the delivery system, DCs and RAW264.7 cells were co-cultured with RA, MLip@Gel, or OR-MLip@Gel in the presence of OVA. PBS and OVA alone served as negative and positive controls, respectively. As shown in Fig. 4A–E and S6, RA and MLip@Gel significantly increased CD80^+^CD86^+^ and MHC II^+^ expression, compared to PBS, indicating effective adjuvant activity. OR-MLip@Gel showed the highest activation, with CD80^+^CD86^+^ reaching 48.66%, nearly double that of OVA group (25.73%). MHC II^+^ expression also increased substantially in DCs (49.46%) and macrophages (41.94%). This enhancement is likely attributable to the liposomes’ promotion of antigen uptake.

To further assess immune activation, cytokines secretion (IL-4, IFN-γ, and TNF-α) were measured by ELISA. Both OVA and OR-MLip@Gel significantly increased cytokines levels compared to PBS, with OR-MLip@Gel inducing the highest levels, especially for IFN-γ (Fig. 4F and G). Similar trends were observed in macrophages, conforming strong immunostimulatory activity.

**Fig. 5:**
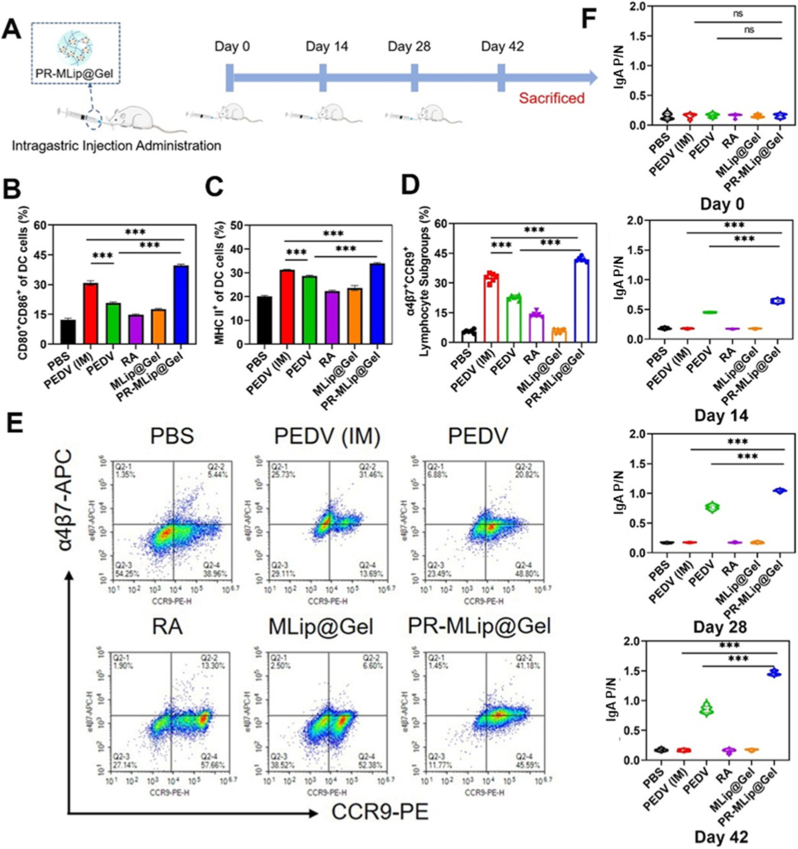
Vaccine *in vivo* immune activation and induction of mucosal immune response. (A) Scheme of vaccine immunization. Expression of CD80^+^CD86^+^ (B), and MHC II^+^ (C) on DCs isolated 96 h after the first immunization (*n* = 3). After immunization. (D) Analysis of the proportion of α4β7^+^CCR9^+^ cells on intestinal epithelial interlymphoid cells. (E) Representative flow cytometry plots of α4β7^+^CCR9^+^ on intestinal epithelial interlymphoid cells in each group. (F) P/N values of IgA in sera of mice (0, 14, 28, and 42 days from top to bottom). Data are presented as mean ± SD, *n* = 6, unless otherwise stated.

*In vivo*, immune activation was evaluated by orally immunizing mice as shown in Fig. 5A. Bone marrow dendritic cells (BMDCs) were isolated 96 h post-immunization, and CD80^+^, CD86^+^ and MHC II^+^ expression were analyzed. PR-MLip@Gel exhibited significantly higher expressions of all markers compared to the oral PEDV and intramuscular (IM) injection groups (Fig. 5B–C, S7). RA and MLip@Gel also increased expression versus PBS group but less effective than PR-MLip@Gel, consistent with *in vitro* results. The results indicate that the PEDV-loaded gel promotes APC maturation and antigen presentation, supporting its potential as an effective oral vaccine platform.

### 2.6 PR-MLip@Gel enhances mucosal immunity and IgA production

PEDV primarily targets small intestinal epithelial cells, causing lesions and underscoring the need for strong mucosal immunity. Secretory IgA (sIgA) neutralizes pathogens at mucosal surfaces.

To confirm this, intestinal epithelial lymphoid cells were isolated from mice 42 days post-immunization, and CCR9^+^ and α4β7^+^ expression were analyzed by flow cytometry. As shown in Fig. 5D and E, PR-MLip@Gel demonstrated 41.87% α4β7^+^CCR9^+^ cells, significantly higher than the oral PEDV group (22.79%). The RA group showed a similar increase, 2.51 times higher than PBS, while MLip@Gel had no significant difference from PBS. These results suggest that RA enhances lymphocyte migration to the intestinal epithelium, strengthening intestinal immune defenses. Retinoic acid drives the differentiation of IgA-secreting plasma cells and up-regulates the gut-homing receptors α4β7 and CCR9 on lymphocytes, thereby facilitating their migration to intestinal immune sites. Our data extend this concept to PEDV-specific immunity by showing a two-fold increase in sIgA compared with oral PEDV alone.

In addition, PEDV-specific IgA levels in serum, feces and intestinal lavage fluids were measured via ELISA. In serum (Fig. 5F), the PR-MLip@Gel and oral PEDV groups showed significantly higher IgA levels than all other groups, whereas PEDV (IM), PBS, RA and MLip@Gel did not differ significantly. Notably, PR-MLip@Gel induced higher IgA levels than oral PEDV group, likely due to enhanced mucus penetration and macrophage targeting. Similar trends were observed in intestinal lavage fluid (Fig. S8) and fecal (Fig. S9) samples, confirming PR-MLip@Gel's capability to induce mucosal immunity. The negligible mucosal IgA response in the PEDV (IM) group further highlights mucosal immune system independence.

### 2.7 The oral vaccine induces a systemic immune response

**Fig. 6:**
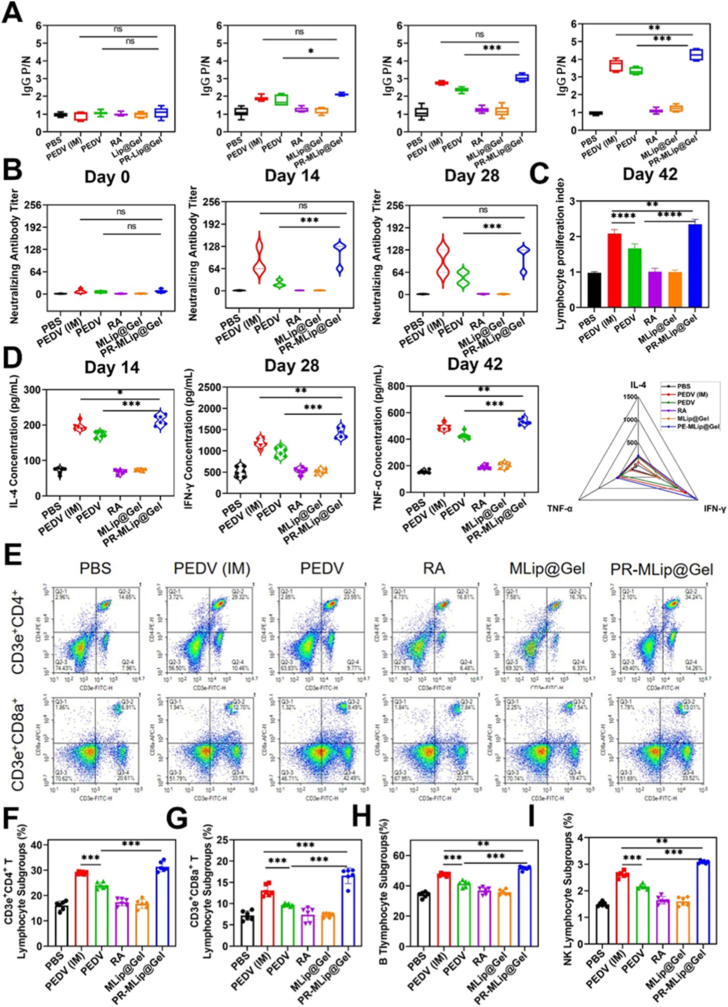
Vaccine-induced systemic immune responses. (A) P/N values of IgG in sera of mice (0, 14, 28, and 42 days from left to right). (B) Serum neutralization titers of mice (14, 28, and 42 days from left to right). (C) Splenic lymphocyte proliferation index. (D) Levels of cytokines (IL-4, IFN-γ, and TNF-α from left to right) secreted by mice after 48 h of spleen cell stimulation. (E) Flow analysis of splenic lymphocytes from mice. (F) Analysis of CD3e^+^CD4^+^ T lymphocyte proportion. (G) Analysis of CD3e^+^CD8^+^ T lymphocyte proportion. (H) Analysis of B220^+^CD19^+^ B lymphocyte proportion. (I) Analysis of CD3e^+^CD49b^+^ NK lymphocytes proportion. Data are expressed as mean ± SD (*n* = 6).

To assess systemic immune responses, PEDV-specific IgG levels in the feces and serum of mice were measured by ELISA (Fig. 6A, S10). At all time points, the PR-MLip@Gel group had higher IgG levels than oral PEDV and PEDV (IM) group, peaking on the 42nd-day post-immunization, indicating a strong and sustained humoral response.

To assess neutralizing antibodies (NAbs) activity, a pseudovirus neutralization test was performed (Fig. 6B). On day 42, PR-MLip@Gel achieved the highest neutralization titer (1:106.67), surpassing oral PEDV (1:48) and PEDV (IM) (1:96), confirming potent NAb induction and protective efficacy.

Spleen lymphocytes collected on day 42 were analyzed for proliferation after ConA stimulation. When re-stimulation with ConA for 48 h, PR-MLip@Gel exhibited the highest proliferation (Fig. 6C), suggesting enhanced immune activation. ELISA analysis of cytokines revealed significantly elevated Th1-type cytokines (IFN-γ and TNF-α) and Th2-type cytokine (IL-4) levels in the PR-MLip@Gel group versus oral and IM groups (Fig. 6D), indicating a strong Th1/Th2 response. Flow cytometry further showed increased levels of CD3e^+^CD4^+^ (31.35%) and CD3e^+^CD8a^+^ (16.57%) T cells in PR-MLip@Gel group compared to the oral PEDV group (24.00%; 9.71%) and PEDV (IM) group (28.89%; 13.06%) (Fig. 6E–G). PR-MLip@Gel induced the highest levels of CD19^+^B220^+^ B cells and CD3e^+^CD49b^+^ NK cells (Fig. 6H–I, and Fig. S11), indicating that PR-MLip@Gel effectively induces both mucosal and systemic immune responses.

### 2.8 PR-MLip@Gel confers protection in immunized piglets

**Fig. 7:**
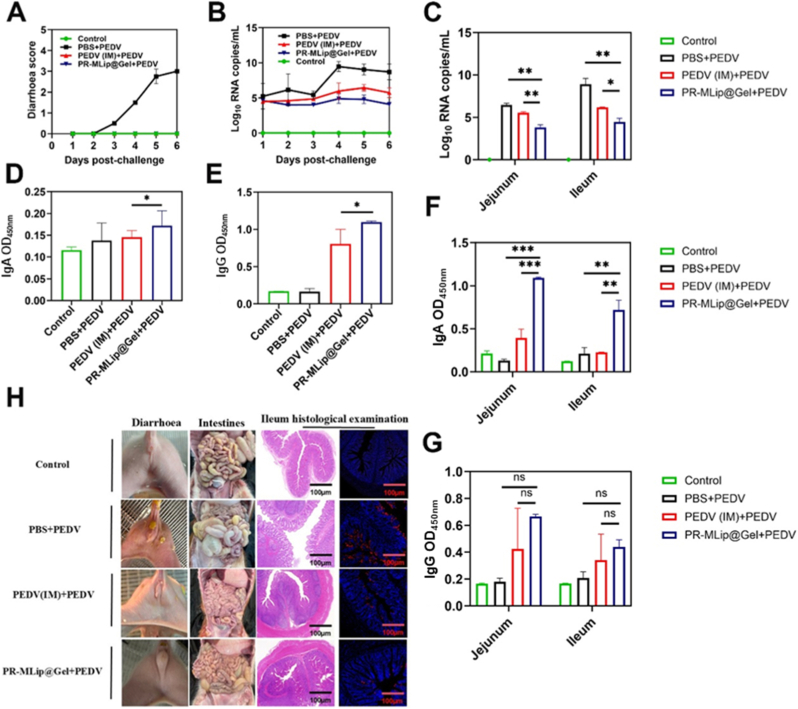
Challenge experiments on immunized piglets. (A) Diarrhea scores after oral administration of PEDV-AH2012/12. Diarrhea scores were: 0 solid, 1 pasty, 2 semiliquid, and 3 liquid. (B) The copy number of rectal swab virus. (C) Jejunal and lleal viral load results at 6 days post-challenge. Serum IgA (D) and IgG (E) levels at 28 days after primary immunization. Intestinal samples were collected to measure the titers of IgA (F) and IgG (G). (H) Clinical, gross, and histopathological lesions in the euthanized piglet intestines at 6 days post-challenge. Representative intestinal sections were examined postmortem, stained with H&E, and analyzed with indirect immunofluorescence. PEDV N was labeled with red fluorescence, and the cell nuclei (blue) were stained with 4,6-diamidino-2-phenylindole (DAPI). Significant differences are indicated as. Data are expressed as mean ± SD (*n* = 3). (For interpretation of the references to colour in this figure legend, the reader is referred to the Web version of this article.)

To verify the protective immunity elicited by vaccination, the challenge experiments were conducted. Piglets in the PBS + PEDV group developed severe diarrhea that peaked on day 6 post-challenge with a mean score of approximately 3 (Fig. 7A), while no obvious diarrhea symptoms were observed in the PR-MLip@Gel + PEDV and PEDV (IM) + PEDV immunized groups. As shown in Fig. 7B, PEDV was undetectable in rectal swabs from the unchallenged control piglets, whereas all challenged animals tested positive. The PBS + PEDV challenged group shed high and persistent viral loads, while the PR-MLip@Gel + PEDV and PEDV(IM) + PEDV immunized groups exhibited only minimal, transient shedding. Furthermore, PR-MLip@Gel + PEDV immunized group displayed a lower viral shedding than the PEDV(IM) + PEDV immunized group. When PEDV RNA loads were quantified in the jejunum and ileum, piglets in the challenged PBS + PEDV group displayed significantly higher viral RNA levels than their vaccinated counterparts. Furthermore, the PR-MLip@Gel + PEDV group exhibited lower viral load in the ileum than that of the PEDV(IM) + PEDV immunized group (Fig. 7C). These results demonstrate that oral PR-MLip@Gel vaccination effectively mitigates diarrhea and reduces the amount of virus excreted in feces in suckling piglets.

Serological analyses performed showed that PR-MLip@Gel + PEDV piglets mounted the highest PEDV-specific IgA (Fig. 7D) and IgG (Fig. 7E) titers in the serum samples, significantly exceeding those of both PBS and PEDV (IM) treated groups. Because PEDV predominantly targets the jejunum and ileum, these intestinal segments were examined in detail. PR-MLip@Gel provoked a strong secretory-IgA response, most notably in the jejunum, where IgA levels were 3.8-fold higher than in the challenged PBS + PEDV group and 1.6-fold higher than in the challenged PEDV (IM) + PEDV group (Fig. 7F). A comparable enhancement was not observed for IgG secretion (Fig. 7G), indicating that in addition to inducing humoral immunity, this oral vaccine also elicited a strong mucosal immunity.

After necropsy, no macroscopic lesions were observed in the small intestines of PR-MLip@Gel and PEDV (IM) vaccinated or unchallenged piglets, whereas the challenged non-immunized group displayed thin, distended and translucent intestinal walls (Fig. 7H). Histopathological examination of ileal sections revealed villus blunting, fragmentation and atrophy in the challenged PBS + PEDV group, while immunized piglets and those unchallenged group retained intact villus architecture. Consistently, an indirect immunofluorescence assay targeting the PEDV nucleocapsid (N) protein detected numerous N-positive enterocytes in the intestines of challenged PBS-treated group but only sparse antigen-positive cells in the challenged vaccinated animals (Fig. 7H).

The above conclusions indicate that piglets immunized with PR-MLip@Gel receive better protection against PEDV infection, which is manifested by alleviated clinical diarrhea symptoms, no observed intestinal lesions, and the lowest viral load in piglets. This protective effect is attributed to PR-MLip@Gel's ability to induce robust mucosal immunity and humoral immunity.

### 2.9 PR-MLip@Gel demonstrates good biocompatibility

Vaccine safety is essential for clinical application. No deaths or abnormal behavior were observed in any group, and body weights in the vaccinated mice increased steadily, suggesting good tolerability (Fig. S12). Histological analysis on day 42 post-immunization further confirmed safety. H&E staining of the small intestine and major organs (heart, liver, spleen, lungs, kidneys) revealed no tissue damage in the PR-MLip@Gel group, indicating excellent biocompatibility (Fig. S13). Moreover, no obvious pathological changes were found in intestine tissues across all groups (Fig. S14).

Interestingly, the PR-MLip@Gel group showed a significant increase in the villus-to-crypt ratio of the small intestine, unlike the PEDV (IM) group and the oral PEDV groups, which exhibited no difference (Fig. S15). This suggests enhanced intestinal epithelial regeneration, contributing to intestinal homeostasis. In conclusion, these findings demonstrate the PR-MLip@Gel's excellent biosafety and its potential for clinical application.

## 3 Conclusion

In this study, we developed a cation-based, mannose-modified liposome gel microsphere oral delivery system (PR-MLip@Gel) capable of eliciting strong mucosal and systemic immune responses against PEDV infection. Cationic liposomes have good biocompatibility, effectively encapsulated PEDV, and readily penetrate the mucus layer to reach intestinal epithelial cells. Mannose modification endowed the liposomes with the ability to target intestinal macrophages, enhancing drug absorption, immune cell activation, antigen presentation, and systemic immune responses. Thiol modification of gel material improves intestinal adhesion, prolongs the antigen retention, and increases the interaction time with the immune system. RA, as an immunopotentiator, promotes the differentiation of plasma cells that secrete IgA, thereby enhancing the mucosal immune effect.

Overall, this oral delivery system demonstrates great potential as a vaccine delivery carrier and an effective mucosal adjuvant.

## CRediT authorship contribution statement

**Zhiwei Li:** Formal analysis, Writing – review & editing. **Baochao Fan:** Formal analysis, Supervision, Writing – review & editing. **Chengcheng Ouyang:** Investigation, Methodology, Software, Validation, Visualization, Writing – original draft, Writing – review & editing. **Mi Hu:** Conceptualization, Software, Writing – review & editing. **Xu Song:** Methodology. **Guoguang Chen:** Investigation. **Yiwen Lou:** Data curation, Visualization. **Huajun Yang:** Investigation. **Dongmei Sun:** Writing – review & editing. **Bin Li:** Funding acquisition, Investigation, Writing – review & editing. **Lili Ren:** Formal analysis, Funding acquisition, Methodology, Writing – review & editing.

## Declaration of competing interest

The authors declare that they have no known competing financial interests or personal relationships that could have appeared to influence the work reported in this paper.

## Data Availability

Data will be made available on request.
