# Supplementary material for: Liposomal hydrogel-based oral vaccine delivery for targeted induction of intestinal mucosal immunity
Source: Mater Today Bio. 2026 Feb 28;37:102975. doi: 10.1016/j.mtbio.2026.102975 (PMC12969131; doi:10.1016/j.mtbio.2026.102975)
Supplement: Multimedia component 1 [file mmc1.docx]

**Supporting Information**

**Liposomal Hydrogel-Based Oral Vaccine Delivery for Targeted Induction of Intestinal Mucosal Immunity**

Zhiwei Li ^a, #^, Baochao Fan ^b, c, #^, Chengcheng Ouyang ^a^, Mi Hu ^b^, Xu Song ^b^, Guoguang Chen ^a^, Yiwen Lou ^a^, Huajun Yang ^a^, Dongmei Sun ^a^, Bin Li ^b, c,^ * & Lili Ren ^a,^ *

a School of Pharmacy, Nanjing Tech University, Nanjing, 211816, China

b Institute of Veterinary Medicine, Jiangsu Academy of Agricultural Sciences, Key Laboratory of Veterinary Biological Engineering and Technology Ministry of Agriculture; Jiangsu Key Laboratory for Food Quality and Safety-State Key Laboratory Cultivation Base of Ministry of Science and Technology, Nanjing 210014, China

c Jiangsu Co-Innovation Center for the Prevention and Control of Important Animal Infectious Disease and Zoonose, Yangzhou University, Yangzhou 225009, PR China;

^#^ These authors contributed equally to this work.

* Corresponding author: To them correspondence should be addressed.

Corresponding Author E-mail: [libinana@126.com](mailto:libinana@126.com) (Bin Li); [renlili@njtech.edu.cn](mailto:renlili@njtech.edu.cn) (Lili Ren).

**Extended Materials and Methods**

**1. Determination of Sulfhydryl Content in Cys-Alg**

The sulfhydryl group content in Cys-Alg was determined using by the 5,5′-dithiobis-2-nitrobenzoic acid (DTNB) reagent. Specifically, Cys-Alg (4.0 mg) was dissolved in water (1 mL), and the pH was adjusted to 4.0 using HCl solution (0.1 M). Subsequently, The Cys-Alg solution was then mixed with an equal volume of DTNB solution (0.3 mg mL^-1^) and reacted incubated in the dark at room temperature for 15 minutes. The absorbance at 412 nm was measured using an ultraviolet (UV) spectrophotometer.

**2. Measurement of Encapsulation Efficiency and Drug Loading of RA-MLip**

The RA-MLip solution was dialyzed in pure water (8000 - 12000 kDa) for 6 hours. Before and after dialysis, RA-MLip solution (0.5 mL) was mixed with an equal volume of methanol to demulsify. RA content was quantified by high-performance liquid chromatography (HPLC) using a KromaSil C18 chromatographic column (5 μm, 250 mm × 4.6 mm). The mobile phase was methanol and 2% acetic acid aqueous solution (92:8, v/v), with an injection (20 μL) volume, flow rate (1.0 mL min^-1^), and detection at 350 nm.

**3. RA-MLip Storage Stability Assay**

To examine the storage stability of RA-MLip was assessed by measuring its, the encapsulation efficiency of RA-MLip was detected every 5 days under four conditions: at 4 °C with light, 4 °C without light, 25 °C with light, and 25 °C without light.

**4. Cytotoxicity Test**

The safety of this oral delivery carrier was verified using the MTT CCK-8 assay. Caco-2 cells, RAW264.7 cells or dendritic cells (DCs) were treated with different concentrations (0, 62.5, 125, 250, 500, and 1000 μg mL^-1^) of MLip, MLip@Gel, and RA-MLip@Gel, and co-incubated at 37 °C for 24 hours.

**5. Determination of Sulfhydryl Content of Cys-Alg**

At 37 °C, Cys-Alg (40 mg) was incubated in SGF (10 mL) or SIF (10 mL). The sulfhydryl content of samples (50 μL, supplemented with the same volume of solvent) was determined by DTNB method every 1 hour.

**6. In Vitro Immune Stimulation**

Ovalbumin (OVA) was used instead of PEDV as the antigen for the cell experiments. Aluminum adjuvant was diluted 1:1.5 with Tris-HCl buffer (pH 8) and mixed with OVA (0.2 mg mL^-1^) at a volume ratio of 1:1. PBS (2 mL), OVA (10 μg mL^-1^, containing aluminum adjuvant), RA (10 μg mL^-1^), MLip@Gel, and liposome gel microspheres loaded with RA and OVA (OR-MLip@Gel) were added to 6-well plates seeded with RAW264.7 cells or DCs. After incubation for 24 hours, the cells were collected and labeled with MHC II-APC, CD80-PE, and CD86-FITC antibodies (Biolegend, USA) in the dark for 30 minutes. The expressions of MHC II^+^, CD80^+^, and CD86^+^ on RAW264.7 cells and DCs were analyzed using flow cytometry. After incubation for 48 hours, supernatant (0.5 mL) from each group was collected, and the contents of cytokines (IFN-γ, IL-4, and TNF-α) were detected by ELISA.


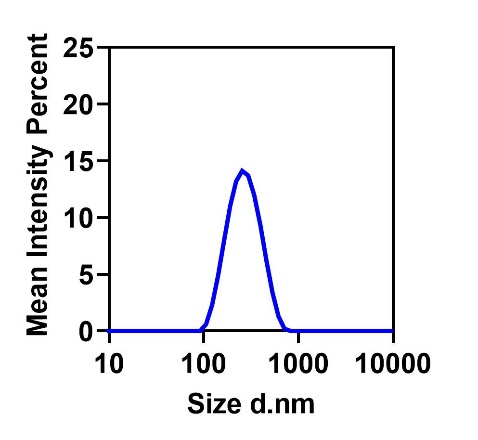


Fig. S1. Particle size distribution of RA-MLip.


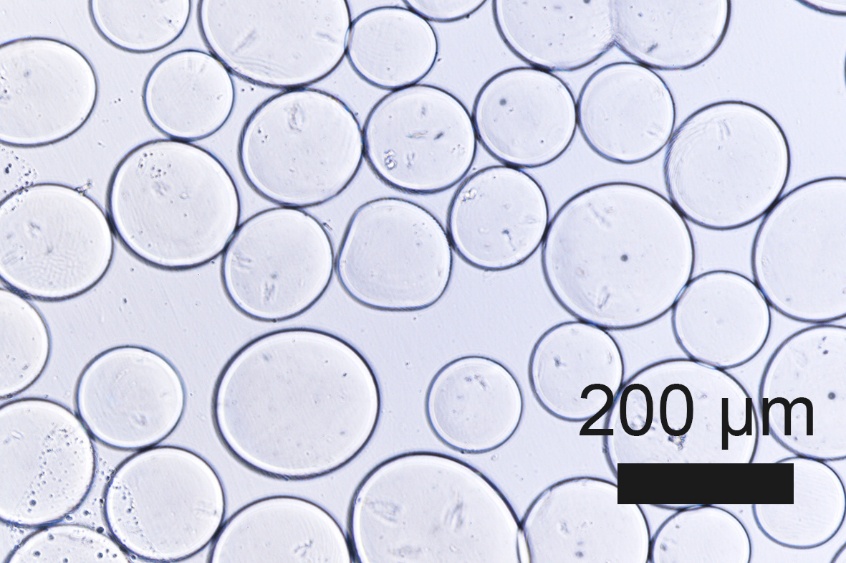


Fig. S2. Image of PR-MLip@Gel under the light field, scale bar: 200 μm).


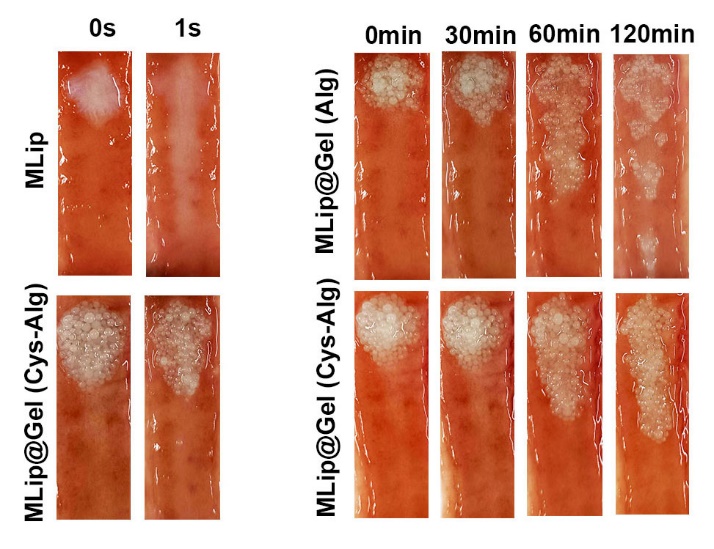


Fig. S3. Adherence of the preparation to fresh porcine small intestine.


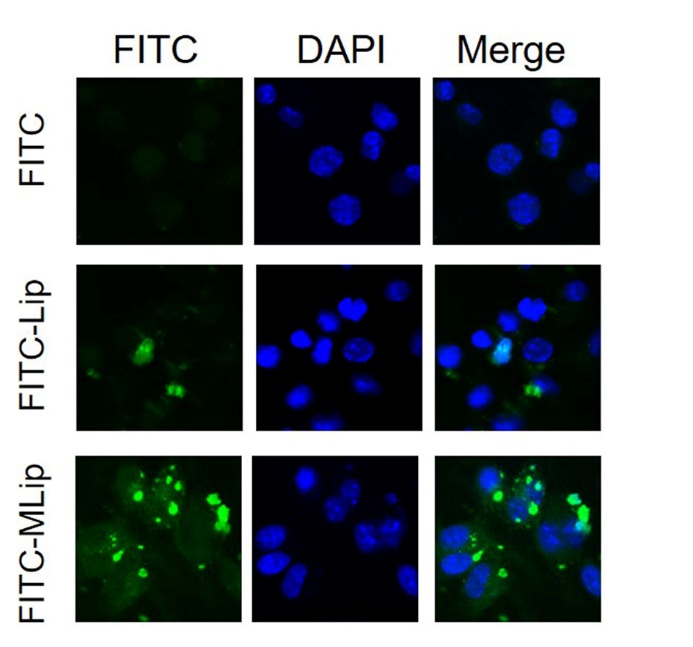


Fig. S4. Confocal images of DCs uptake (Magnification: 40×).


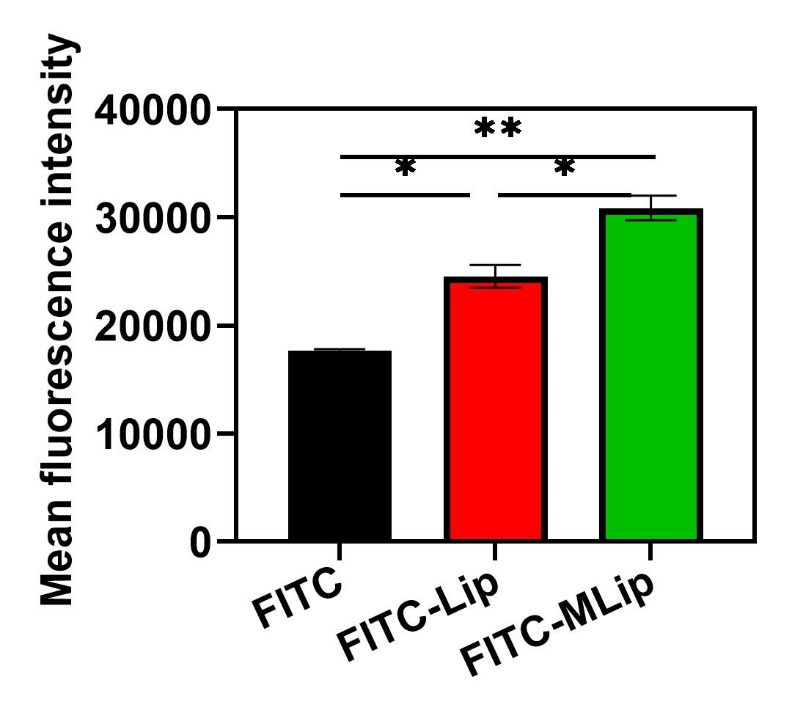


Fig. S5. Fluorescence quantitative analysis of uptake by DCs.


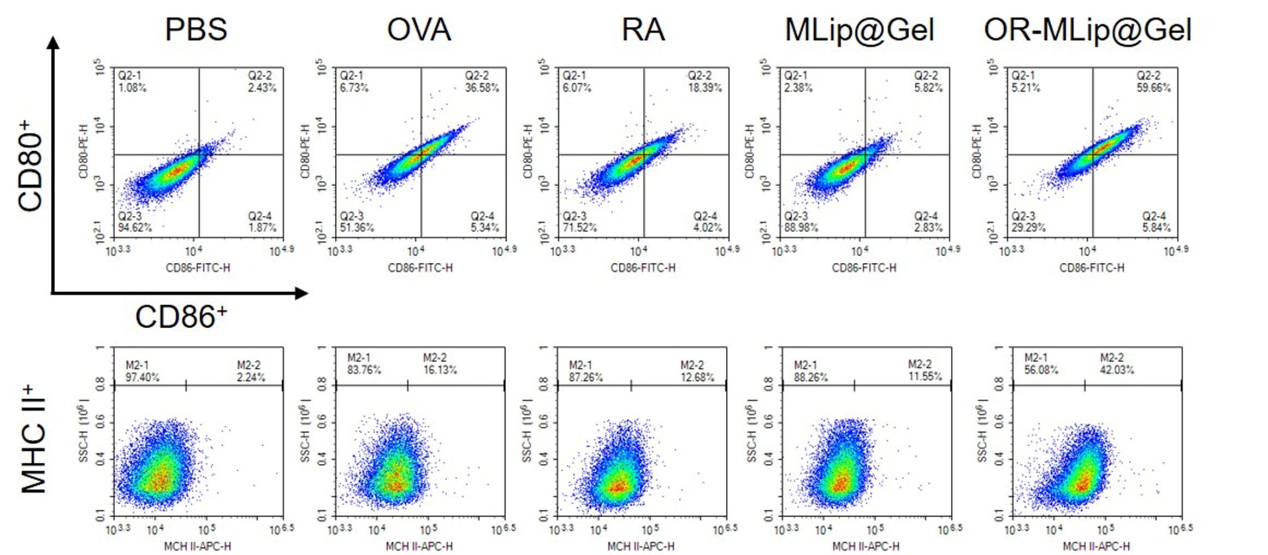


Fig. S6. Representative flow cytometry plot of CD80^+^CD86^+^ and MHC II^+^ on RAW264.7 cells.


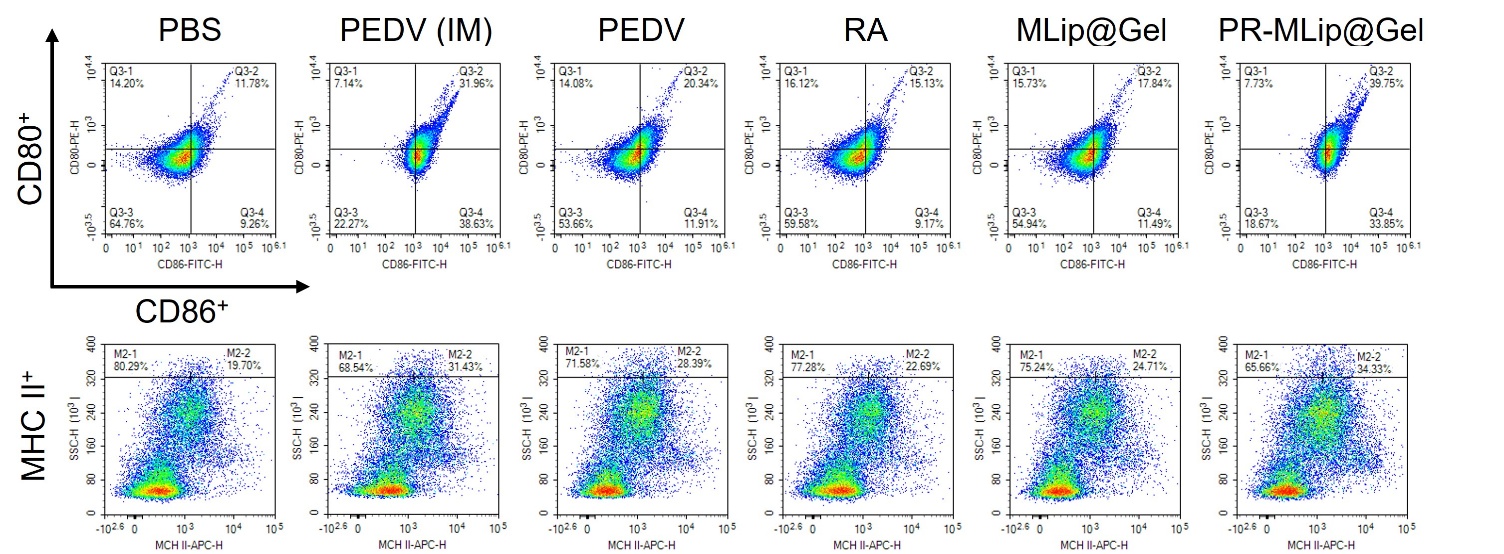


Fig. S7. Representative flow cytometry plot of CD86^+^CD80^+^ and MHC II^+^ on DCs.


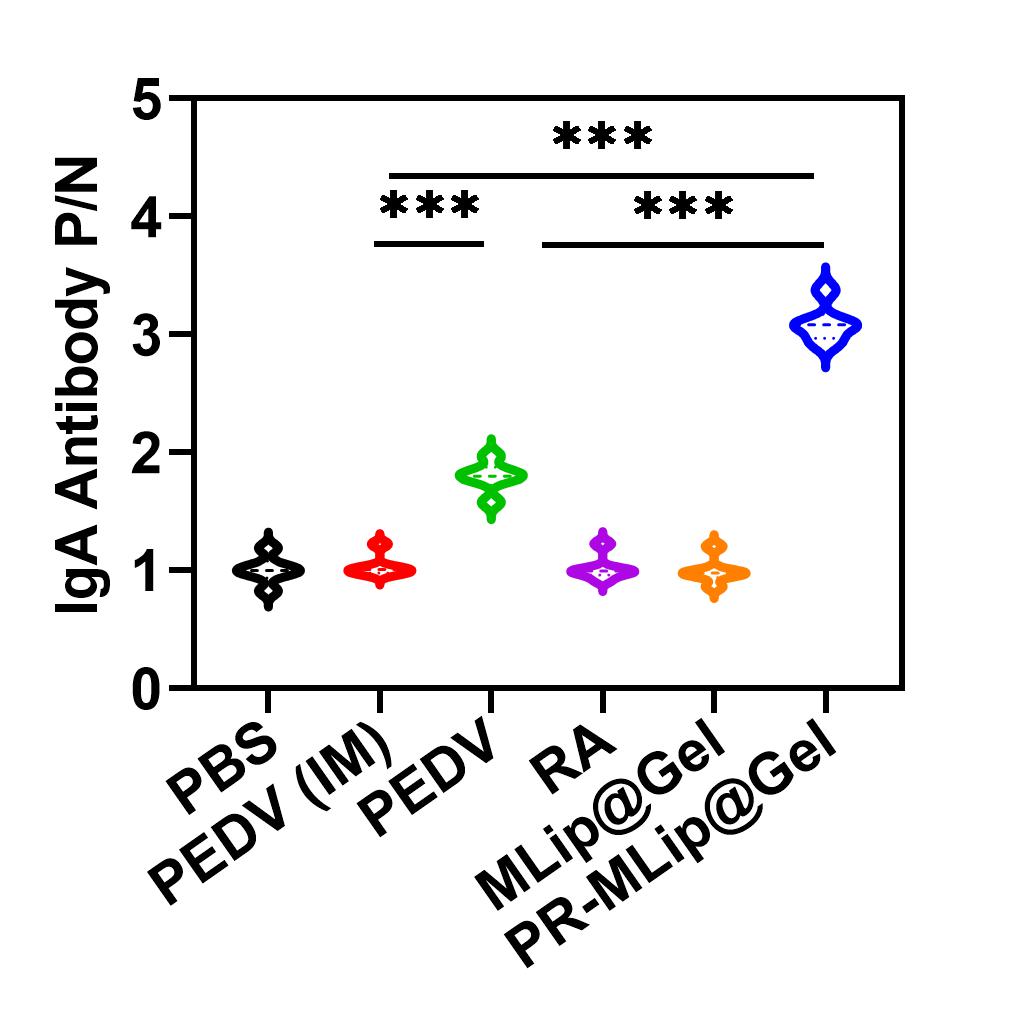


Fig. S8. P/N values of IgA antibodies in intestinal lavage fluid of mice.


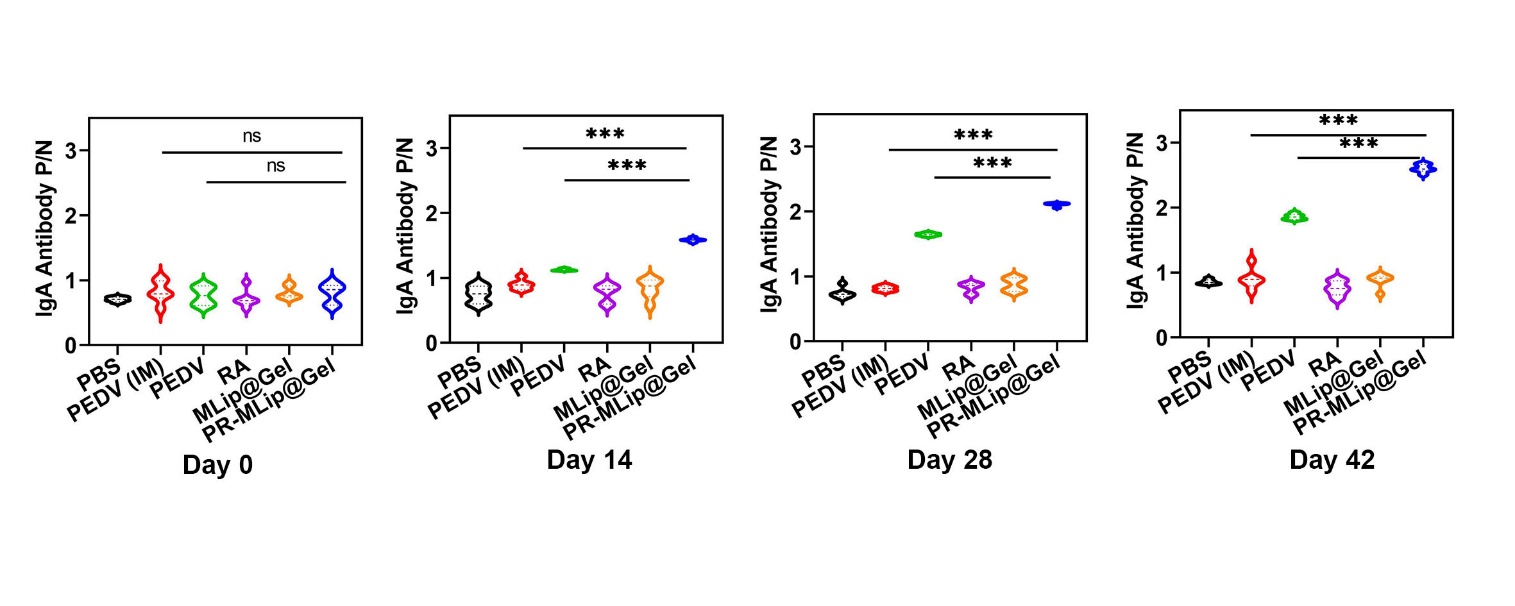


Fig. S9. P/N values of IgA antibodies in fecal samples of mice (0, 14, 28, and 42 days from left to right).


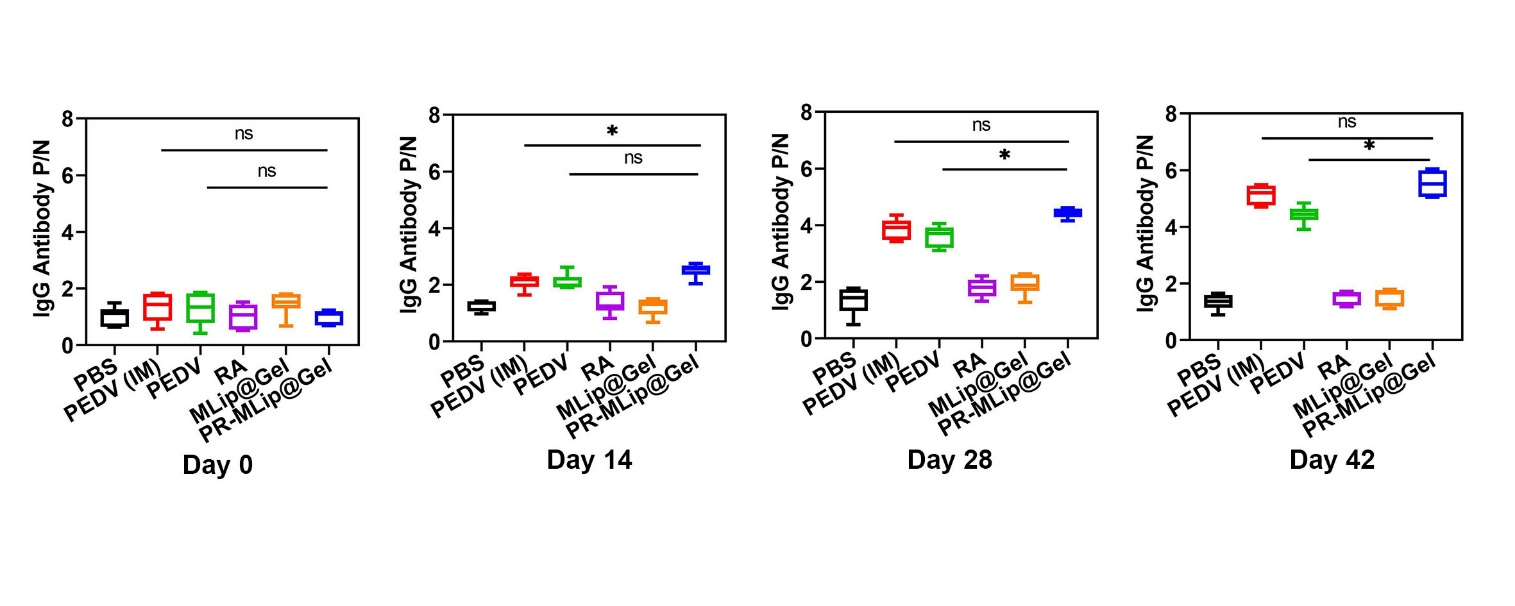


Fig. S10. P/N values of IgG antibodies in fecal samples of mice (0, 14, 28, and 42 days from left to right).


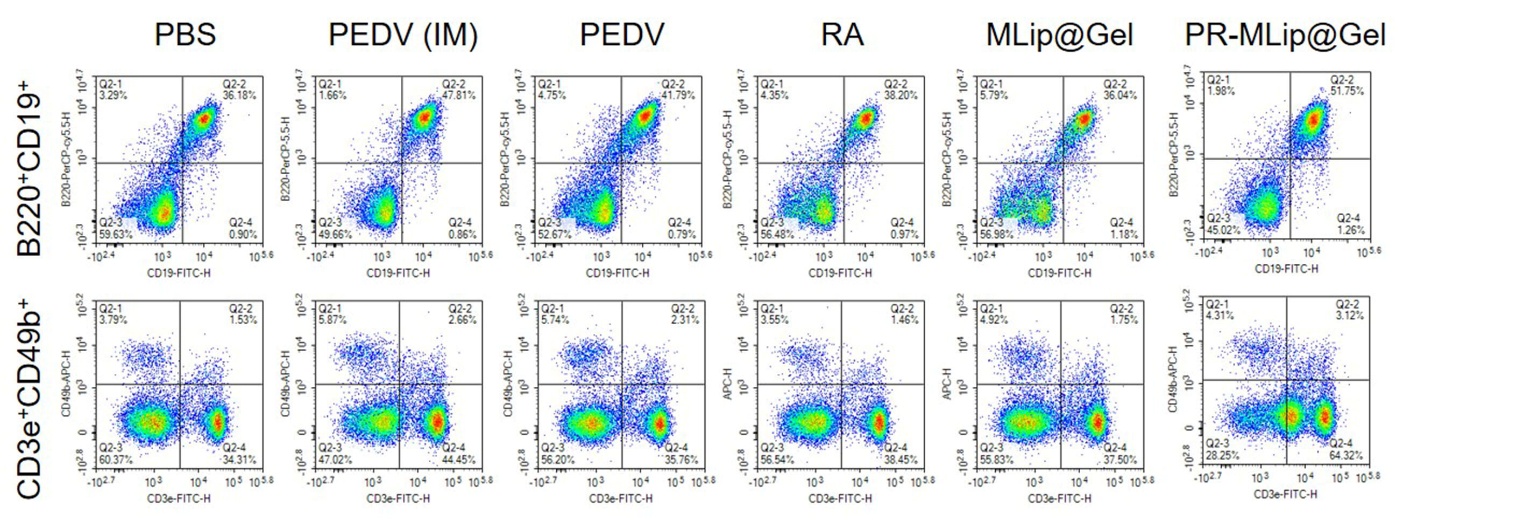
 Fig. S11. Flow analysis of splenic lymphocytes from mice.


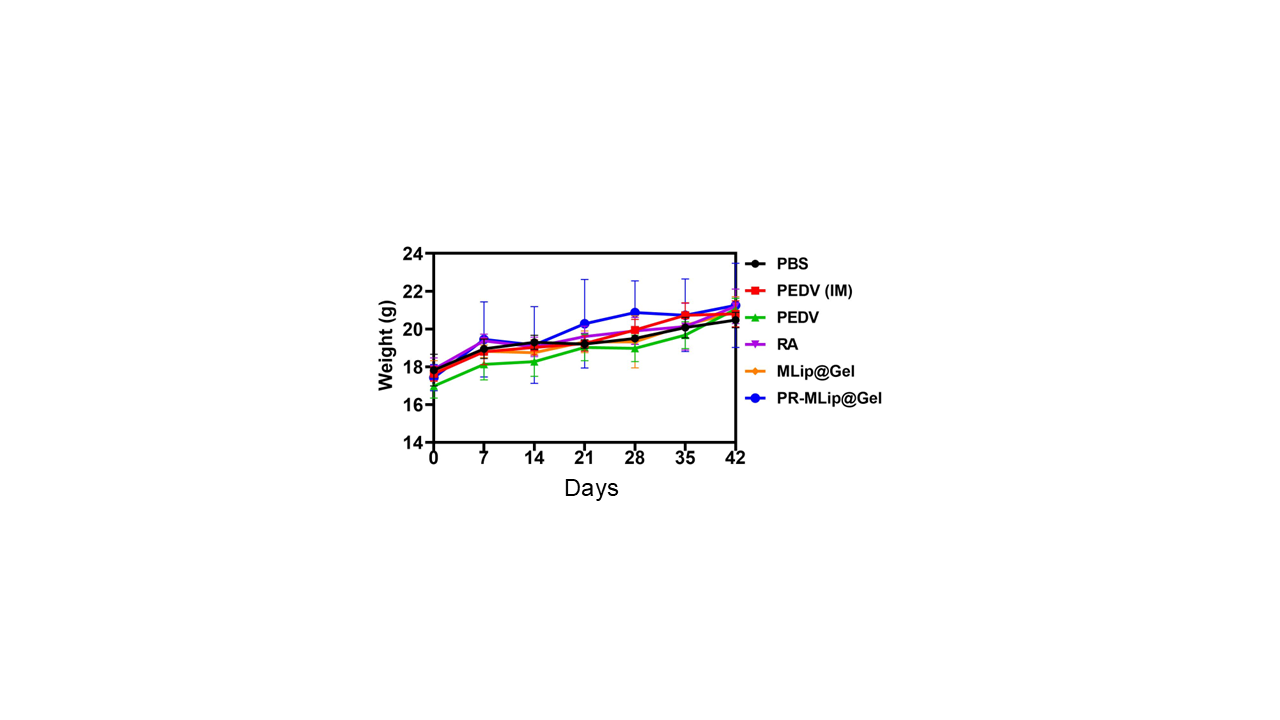


Fig. S12. Curves of changes in body weight of mice during immunization.


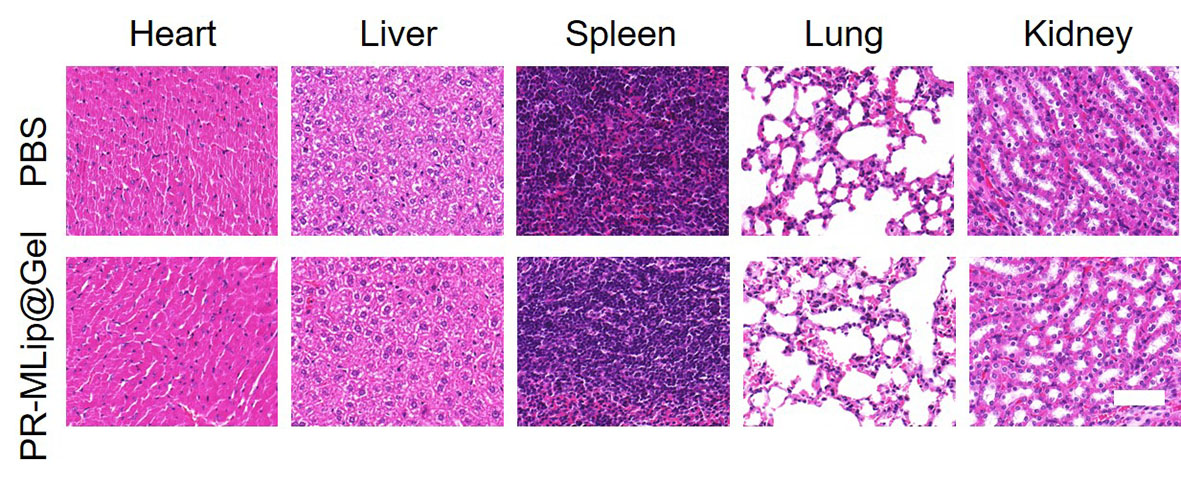


Fig. S13. After immunization, H&E staining of heart, liver, spleen, lung, and kidney was performed (Scale bar: 200 μm).


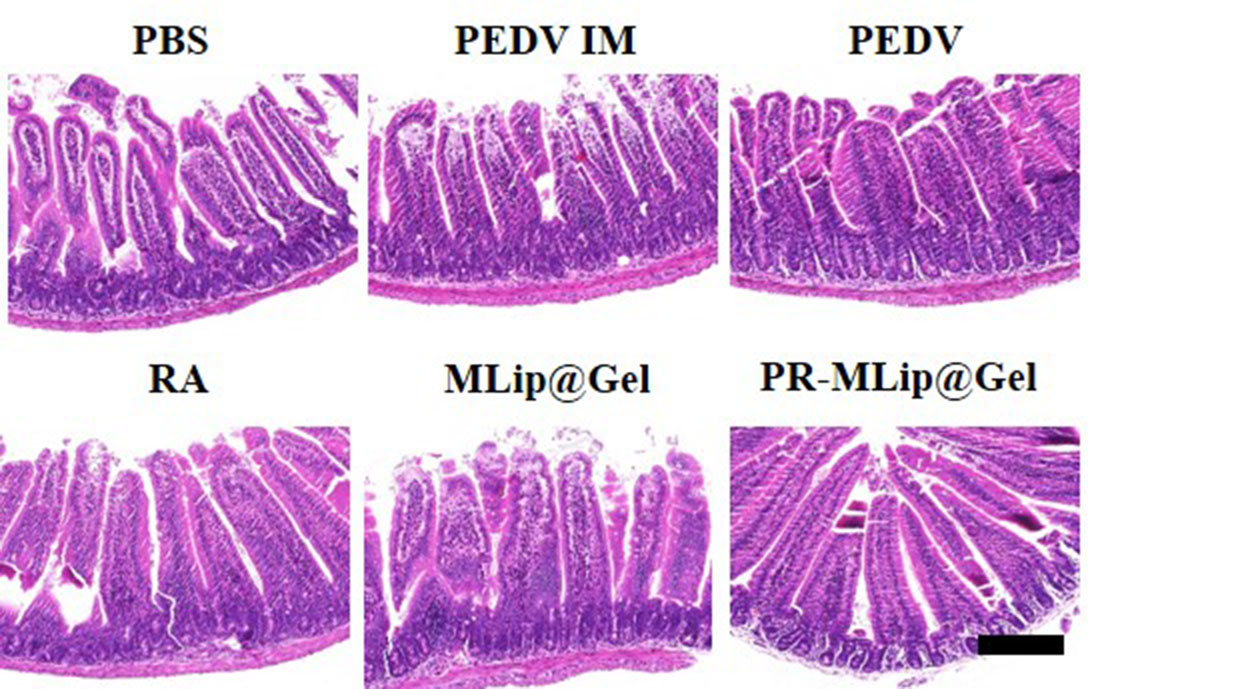


Fig. S14. After immunization, H&E staining of small intestine was performed (Scale bar: 200 μm).


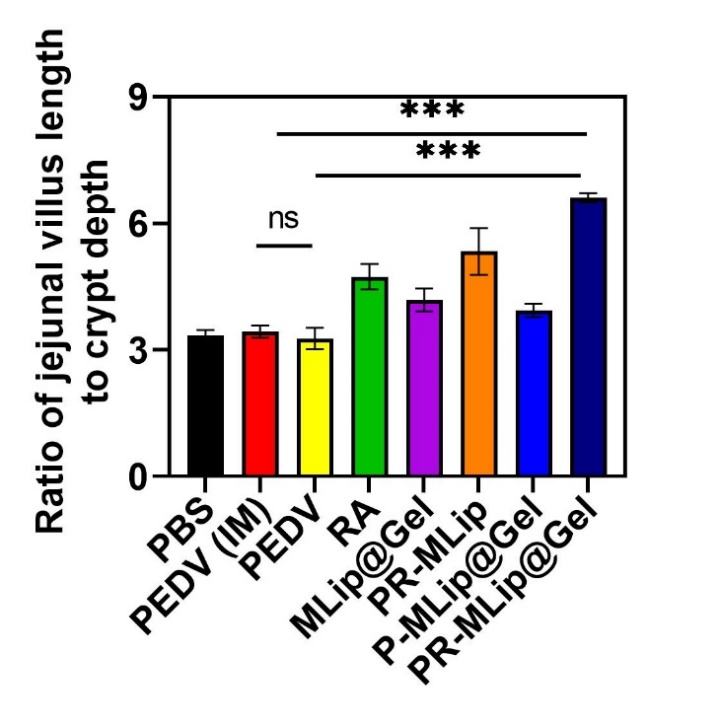


Fig. S15. After immunization, the ratio of small intestinal villus length to crypt depth was determined in each group.
